# Neutralization of oxidized phospholipids attenuates age‐associated bone loss in mice

**DOI:** 10.1111/acel.13442

**Published:** 2021-07-19

**Authors:** Michela Palmieri, Maria Almeida, Intawat Nookaew, Horacio Gomez‐Acevedo, Teenamol E. Joseph, Xuchu Que, Sotirios Tsimikas, Xiaoli Sun, Stavros C. Manolagas, Joseph L. Witztum, Elena Ambrogini

**Affiliations:** ^1^ Division of Endocrinology and Metabolism Center for Osteoporosis and Metabolic Bone Diseases and Center for Musculoskeletal Disease Research University of Arkansas for Medical Sciences and Central Arkansas Veterans Healthcare System Little Rock AR USA; ^2^ Department of Biomedical Informatics University of Arkansas for Medical Sciences Little Rock AR USA; ^3^ Division of Endocrinology and Metabolism Department of Medicine University of California San Diego La Jolla CA USA; ^4^ Department of Medicine Division of Cardiology University of California San Diego La Jolla CA USA

**Keywords:** aging and bone, osteoblasts, oxidized phospholipids, Wnt signaling

## Abstract

Oxidized phospholipids (OxPLs) are pro‐inflammatory molecules that affect bone remodeling under physiological conditions. Transgenic expression of a single‐chain variable fragment (scFv) of the antigen‐binding domain of E06, an IgM natural antibody that recognizes the phosphocholine (PC) moiety of OxPLs, increases trabecular and cortical bone in adult male and female mice by increasing bone formation. OxPLs increase with age, while natural antibodies decrease. Age‐related bone loss is associated with increased oxidative stress and lipid peroxidation and is characterized by a decline in osteoblast number and bone formation, raising the possibility that increased OxPLs, together with the decline of natural antibodies, contribute to age‐related bone loss. We show here that transgenic expression of E06‐scFv attenuated the age‐associated loss of spinal, femoral, and total bone mineral density in both female and male mice aged up to 22 and 24 months, respectively. E06‐scFv attenuated the age‐associated decline in trabecular bone, but not cortical bone, and this effect was associated with an increase in osteoblasts and a decrease in osteoclasts. Furthermore, RNA‐seq analysis showed that E06‐scFv increased Wnt10b expression in vertebral bone in aged mice, indicating that blocking OxPLs increases Wnt signaling. Unlike age‐related bone loss, E06‐scFv did not attenuate the bone loss caused by estrogen deficiency or unloading in adult mice. These results demonstrate that OxPLs contribute to age‐associated bone loss. Neutralization of OxPLs, therefore, is a promising therapeutic target for senile osteoporosis, as well as atherosclerosis and non‐alcoholic steatohepatitis (NASH), two other conditions shown to be attenuated by E06‐scFv in mice.

## Graphical abstract

During aging, oxidized phospholipids (OxPL) increase, while IgM natural antibodies, which recognize OxPL, decrease. Transgenic expression of E06‐scFv, an IgM natural antibody fragment that recognizes the phosphocholine (PC) moiety of OxPL, attenuates the age‐related bone loss and increases Wnt10b expression in the bone of aged mice. OxPL contribute to age‐associated bone loss, and neutralization of OxPL is a promising therapeutic target for senile osteoporosis.

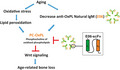

**Abbreviations**
Anti‐Pc: anti phosphocholine
B. Pm: bone perimeter
BFR: bone formation rate
BMD: bone mineral density
BS: bone surface
BV/TV: bone volume /total volume
DXA: dual‐energy X‐ray absorptiometry
LDLR‐KO: low density lipoprotein receptor knockout
MAR: mineral apposition rate
MS: mineralized surface
N. Ob: osteoblast number
Ob.S: osteoblast surface
Oc.N: osteoclast number
Oc.S: osteoclast surface
OS: oxidative stress
OVX: Ovariectomy
OxPLs: oxidized phospholipids
PC: phosphocholine
qPCR: quantitative polymerase chain reaction
ROS: reactive oxygen species
Sc‐Fv: single‐chain variable fragment
Tb: trabecular
WT: wild type

## 1 INTRODUCTION

Phospholipids containing the phosphocholine head group (PC) are among the most common phospholipids and integral components of all cellular membranes. Phospholipids containing polyunsaturated fatty acids are susceptible to lipid peroxidation caused by reactive oxygen species (ROS). Such oxidized PC‐containing phospholipids (referred to as OxPLs) are ubiquitous in many inflammatory states and are present on the surface of apoptotic cells and oxidized low‐density lipoproteins (OxLDL), but not on viable cells and native LDL (Binder et al., 2016). OxPLs are recognized by the IgM natural antibody E06, which is produced by B‐1 lymphocytes and can block many of the pro‐inflammatory properties of OxPLs (Palmieri et al., 2021).

Lipid peroxidation and OxPLs increase with age (Barrera et al., 2018; Liu et al., 2013), while the B‐1 cells that produce natural antibodies against OxPLs decline with age in humans (Griffin et al., 2011; Holodick & Rothstein, 2015; Holodick et al., 2016; Rothstein, 2016). Similarly, anti‐phosphocholine (anti‐PC) IgM antibodies, of which E06 is an important component, decrease in 22‐ to 26‐month‐old mice as compared to 6‐ to 7‐month‐old mice (Ambrogini et al., 2018). The increase in OxPLs, combined with the decline of innate immunity, may contribute to multiple age‐associated conditions such as atherosclerosis (Binder et al., 2016; Que et al., 2018), cardiovascular disease (Byun et al., 2017; Capoulade et al., 2018; Tsimikas et al., 2012), steatohepatitis (Sun et al., 2019), macular degeneration (Handa et al., 2015; Suzuki et al., 2012), and Alzheimer's dementia (Ademowo et al., 2017; Binder et al., 2016).

We have previously shown that transgenic expression of a single‐chain variable fragment of E06 (E06‐scFv), which binds to the PC head group of OxPL (but not native phospholipids), protected against high‐fat diet‐induced bone loss in LDL‐receptor knockout (LDLR‐KO) mice (Ambrogini et al., 2018). In addition, E06‐scFv, in a dose‐dependent manner, increased trabecular and cortical bone mass in chow‐fed C57BL/6 male and female mice at 6 months of age, indicating that lipid peroxidation adversely affects bone remodeling even under physiological conditions (Palmieri et al., 2021). The increase in bone mass produced by E06‐scFv was due to increased osteoblast number and bone formation rate in both the trabecular compartment and the endocortical surfaces of long bones and decreased osteoblast apoptosis (Palmieri et al., 2021). E06‐scFv did not affect osteoclast number in trabecular or cortical bone of the femur, but decreased osteoclast number in the trabecular bone of the vertebra (Ambrogini et al., 2018; Palmieri et al., 2021) and decreased osteoclastogenesis in *ex*‐*vivo* cultures (Palmieri et al., 2021).

Age‐related bone loss is characterized by a decline in osteoblast number and bone formation (Almeida et al., 2007) and is associated with increased oxidative stress and generation of lipid peroxidation products (Almeida et al., 2009; Manolagas, 2010), which in turn decrease Wnt signaling—a seminal stimulus for osteoblastogenesis (Liu et al., 2016).

In view of the evidence that OxPLs suppress osteoblast number and increase with age, while anti‐PC IgM decrease with age, we investigated whether E06‐scFv protects against age‐related bone loss in female and male mice. Because oxidative stress (OS) is involved in the bone loss caused by sex‐steroid deficiency (Almeida et al., 2007, 2017; Lean et al., 2003; Manolagas, 2010) and elevated OS markers are found in models of unloading‐induced bone loss (Morikawa et al., 2013), we also examined whether OxPLs might contribute to bone loss in these conditions.

We found that the E06‐scFv transgene attenuates the age‐associated loss of trabecular, but not cortical, bone in both female and male mice and has no effect on the bone loss caused by ovariectomy (OVX) or unloading. The attenuation of age‐related trabecular bone loss was accompanied by increased osteoblast number and decreased osteoclast number throughout life. Wnt10b expression was increased in the bone of aged E06‐scFv transgenic mice, suggesting that neutralization of OxPLs increases bone mass by affecting Wnt signaling.

## 2 RESULTS

### 2.1 E06‐scFv attenuates the age‐related decrease in bone mineral density

**FIGURE 1:**
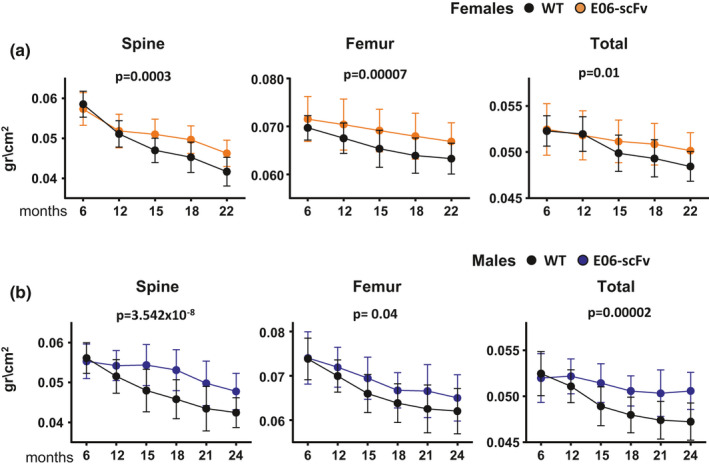
E06‐scFv attenuates age‐related decline in BMD in both sexes. Determinations of femoral, spinal, and total bone mineral density by DXA in (a) females between 6 and 22 months of age [WT mice *n* = 22–25; E06‐scFv *n* = 14–26] and in (b) males between 6 and 24 months of age [WT mice *n* = 24–32; E06‐scFv *n* = 23–26]. Data are shown as mean and standard deviation. Adjusted *p*‐values were calculated by repeated measures using two‐way ANOVA

Female and male WT and homozygous E06‐scFv transgenic littermates were aged up to 22 and 24 months, respectively. Bone mineral density (BMD) was measured with dual‐energy X‐ray absorptiometry (DXA) every 3 months, starting at 6 months of age, until euthanasia. At 6 months of age, BMD did not differ between WT and E06‐scFv transgenic mice at any site. The E06‐scFV transgene, however, attenuated the subsequent age‐related loss of spinal, femoral, and total BMD in both female and male mice (Figure 1A,B).

### 2.2 E06‐scFv attenuates age‐related loss of trabecular bone

**FIGURE 2:**
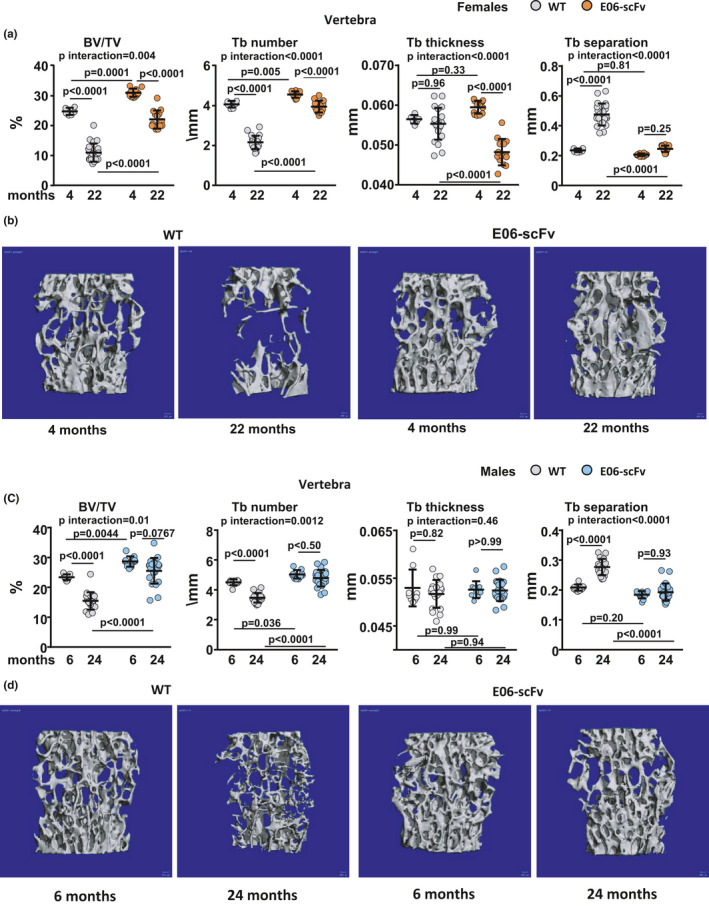
E06‐scFv attenuates the age‐associated decline in vertebral trabecular bone in female and male mice. a, Quantification of trabecular bone architecture by micro‐CT in the vertebrae of 4‐ and 22‐month‐old WT and E06‐scFv transgenic female mice [WT *n* = 6–22; E06‐scFv *n* = 12–14]. b, Lateral images of vertebral trabecular bone (one representative image for each group of female mice). c, Quantification of trabecular bone architecture by micro‐CT in the vertebrae of 6‐ and 24‐month‐old WT and E06‐scFv transgenic male mice [WT *n* = 9–19; E06‐scFv *n* = 10–22]. d, Lateral images of vertebral trabecular bone (one representative image for each group of male mice). Data are shown as individual values including mean and standard deviation. Data analyzed by two‐way ANOVA, the shown p‐values were adjusted using the Sidak's multiple comparison procedure. BV/TV=bone volume /total volume; Tb=trabecular

We reported that E06‐scFv increases trabecular bone at 6 months of age in both female and male transgenic mice fed a chow diet (Palmieri et al., 2021). In the present study, micro‐CT analysis of the vertebra (Figure 2A) and femur (Supplementary Figure 1A) in 4‐month‐old female mice confirmed that E06‐scFv increased trabecular bone at both sites by increasing the trabecular number. Strikingly, E06‐scFv attenuated the age‐associated loss of trabecular bone in the vertebrae of female mice (Figure 2A). In fact, WT mice lost 55.8% of vertebral trabecular bone between 4 and 22 months of age, while the E06‐scFv transgenic mice lost 28.9% of bone at the same site (*p* interaction by two‐way ANOVA = 0.004). E06‐scFv attenuated the decrease in trabecular number and the increase in trabecular separation. Aged E06‐scFv transgenic female mice, however, had reduced trabecular thickness compared with their young controls and compared with aged WT mice.

The majority of trabecular bone at the distal femur is lost early in life, particularly in female C57BL/6 mice. Indeed, in WT female mice, the trabecular bone at the femoral metaphysis was already very low at 4 months, and we could not detect a loss with aging (Supplementary Figure 1A). In E06‐scFv mice, however, the transgene caused a further increase in trabecular bone between 4 and 22 months.

Confirming our previous findings (Palmieri et al., 2021), E06‐scFv also increased vertebral and femoral trabecular bone at 6 months of age in male mice (Figure 2 C and Supplementary Figure 1B). Similar to females, E06‐scFv attenuated the age‐associated loss of trabecular bone in the vertebrae of male mice (Figure 2C). In fact, while WT mice lost 33.8% of vertebral trabecular bone between 6 and 24 months of age, E06‐scFv transgenic males lost only 10.6% of bone at the same site (*p* interaction by two‐way ANOVA 0.01). E06‐scFv attenuated the decrease in trabecular number and the increase in trabecular separation but did not affect trabecular thickness. In the trabecular bone of the femur, WT mice lost 68.3% of trabecular bone, while E06‐scFv mice lost 40.3% (Supplementary Figure 1B). However, the rate of bone loss did not differ between the genotypes.

### 2.3 E06‐scFv does not affect age‐related cortical bone loss

**FIGURE 3:**
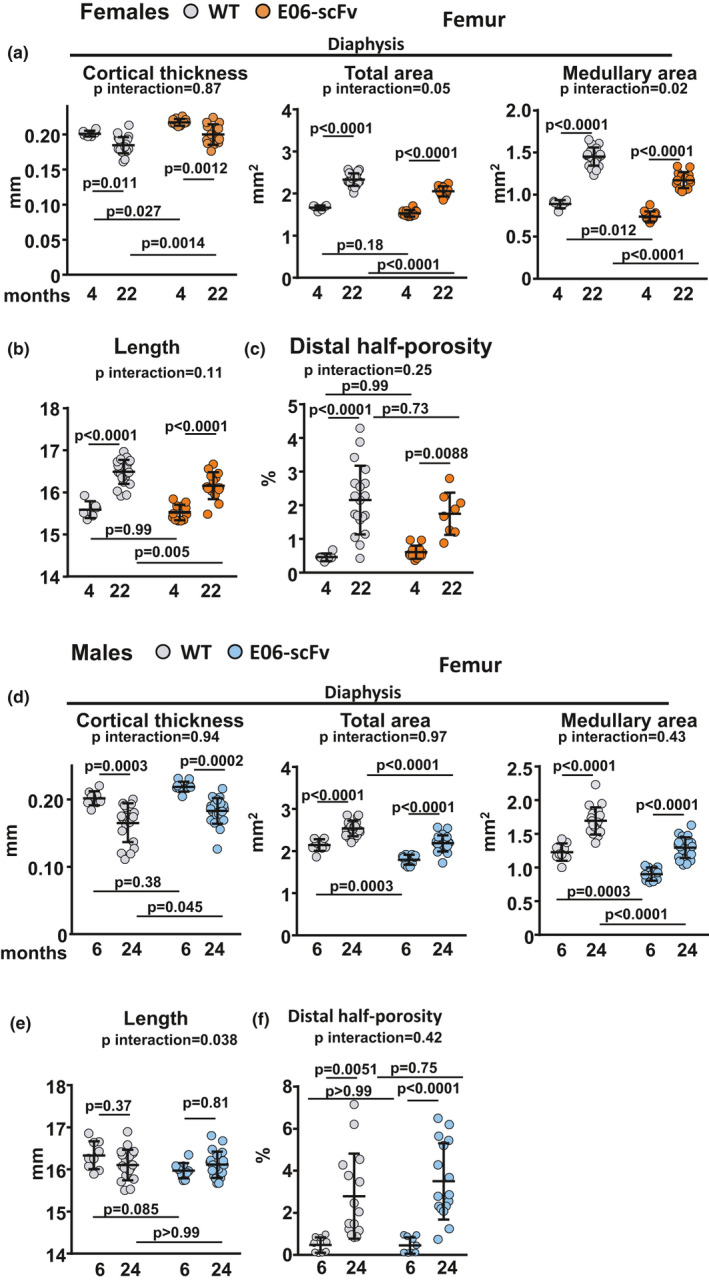
E06‐scFv does not attenuate age‐related cortical bone loss in female or male mice. a‐c, Quantification of femoral cortical bone architecture by micro‐CT in WT and E06‐scFv transgenic female mice. a, Femoral diaphysis architecture [WT *n* = 6–22; E06‐scFv *n* = 12–14]. b, Femoral length [WT *n* = 6–22; E06‐scFv *n* = 12–14]. c, Cortical porosity [WT *n* = 6–19; E06‐scFv *n* = 8–12]. d‐f, Quantification of femoral cortical bone architecture by micro‐CT in WT and E06‐scFv transgenic male mice. d, Femoral diaphysis architecture [WT *n* = 9–19; E06‐scFv *n* = 10–22]. e, Femoral length [WT *n* = 9–19; E06‐scFv *n* = 10–20]. f, Cortical porosity [WT *n* = 9–15; E06‐scFv *n* = 10–16]. Data are shown as individual values including mean and standard deviation. Data analyzed by two‐way ANOVA; the shown *p*‐values were adjusted using Sidak's multiple comparison procedure

We next investigated the effect of E06‐scFv on cortical bone of the femur. In 4‐month‐old female mice, E06‐scFv increased cortical thickness at the diaphysis by 8.0% (Figure 3A), similar to what was previously reported in 6‐month‐old mice (Palmieri et al., 2021). The increase in cortical thickness was due to a decrease in medullary area. Between 4 and 22 months of age, WT and E06‐scFv female mice lost a similar amount of cortical thickness, 8.1% and 10.8%, respectively (Figure 3A). However, the increase in total area, which reflects periosteal apposition with age, was attenuated by the E06‐scFv transgene in females. Likewise, the expansion of the medullary area that occurs with age was attenuated by the transgene. E06‐scFv did not impact the increase in femoral length (Figure 3B) or the age‐associated increase in cortical porosity (Figure 3C).

The analysis of the cortical bone in male mice showed similar results. In 6‐month‐old mice, E06‐scFv increased cortical thickness by 8.5% at the diaphysis (Figure 3D), due to a decrease in medullary area similar to what was reported previously (Palmieri et al., 2021). As observed in females, expression of E06‐scFv did not attenuate the age‐induced loss of cortical thickness (Figure 3D). Specifically, WT mice lost 18.1% of cortical thickness and E06‐scFv transgenic mice lost 16.3% of cortical thickness between 6 and 24 months of age. In contrast to females, the transgene did not impact the expansion of total area or medullary area with age. In addition, E06‐svFv did not affect femoral length (Figure 3E) or the age‐associated increase in cortical porosity (Figure 3F).

### 2.4 Old E06‐scFv transgenic mice have increased osteoblasts and activity and decreased osteoclasts in vertebral bone

**FIGURE 4:**
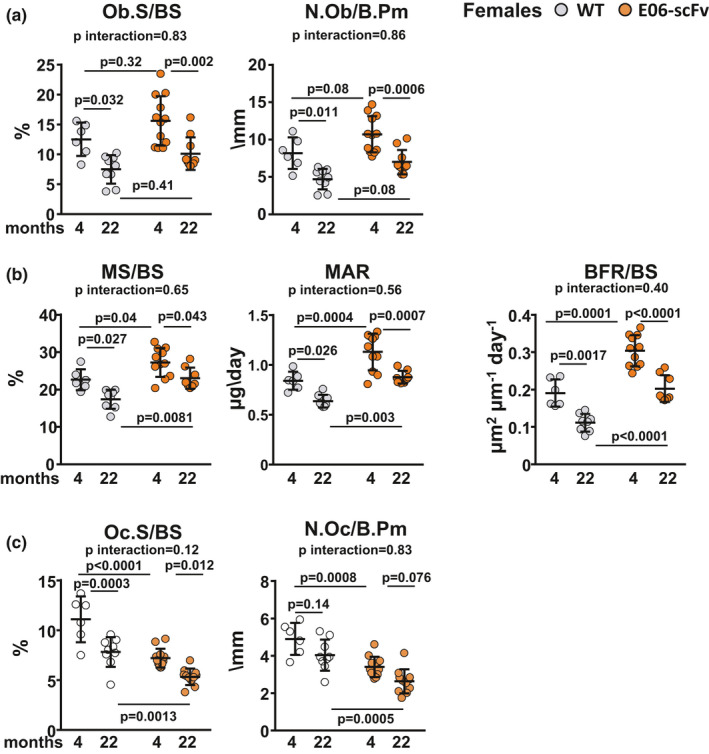
E06‐scFv affects vertebral trabecular bone mass in female mice by increasing osteoblasts and decreasing osteoclasts. Analysis of the histomorphometric parameters in the vertebral bone of female mice. a, Osteoblast surface and osteoblast number [WT *n* = 6–9; E06‐scFv *n* = 10–12]. b, Dynamic measurements [WT *n* = 6–8; E06‐scFv *n* = 8–11]. c, Osteoclast surface and number [WT *n* = 6–9; E06‐scFv *n* = 10–12]. Data are shown as individual values including mean and standard deviation. Data analyzed by two‐way ANOVA; the shown *p*‐values were adjusted using Sidak's multiple comparison procedure. Ob.S = osteoblast surface; BS = bone surface; N. Ob = osteoblast number; B. Pm = bone perimeter; MS = mineralized surface; MAR = mineral apposition rate; BFR = bone formation rate; Oc.S = osteoclast surface; Oc.N = osteoclast number

Histomorphometric analysis of the vertebrae of females confirmed that, in young mice, E06‐scFv increased osteoblast number and bone formation and decreased osteoclast number (Figure 4A‐C), as previously reported (Palmieri et al., 2021). In WT mice, aging caused a 40.1% decrease in osteoblast surface, 42.4% decrease in osteoblast number, 23.2% decrease in mineralized surface, 24.3% decrease in mineral apposition rate, 41.7% decrease in bone formation rate, 29.6% decrease in osteoclast surface, and 17.6% decrease in osteoclast number. These results confirm previous reports indicating a decline in osteoblast number, bone formation, and osteoclast surface in the trabecular bone of aged mice (Almeida et al., 2007). In aged E06‐scFv transgenic mice, we observed a similar decrease in osteoblast parameters. Specifically, we detected a 35.2% decrease in osteoblast surface, 34.6% decrease in osteoblast number, 15.5% decrease in mineralized surface, 22.5% decrease in mineral apposition rate, and 33.3% decrease in bone formation rate compared with young transgenic mice. Thus, although the static and dynamic indices of osteoblastogenesis declined with age in both groups, osteoblast surface and osteoblast number remained 35.0% and 48.7% higher, respectively, in aged E06‐scFv transgenic mice compared with aged WT mice (Figure 4A,B). Similarly, mineralized surface, mineral apposition rate, and bone formation rate remained 32.4%, 37.8%, and 82.1% higher, respectively, in E06‐scFv aged transgenic mice.

In both WT and aged E06‐scFv transgenic mice, we observed a 29.6% and 28.1% decline in osteoclast surface and 38.5% and 26.9% decline in osteoclast number, respectively (Figure 4C). Nonetheless, aged E06‐scFv transgenic mice maintained a 33.9% lower osteoclast surface and 38.5% lower osteoclast number compared with aged WT mice.

### 2.5 E06‐scFv does not affect ovariectomy‐ or unloading‐induced bone loss

Oxidative stress has been noted in other models of bone loss, such as ovariectomy (OVX) (Almeida et al., 2007, 2017; Lean et al., 2003; Manolagas, 2010) and unloading (Morikawa et al., 2013). Therefore, we sought to determine whether OxPLs contribute to bone loss in these models.

**FIGURE 5:**
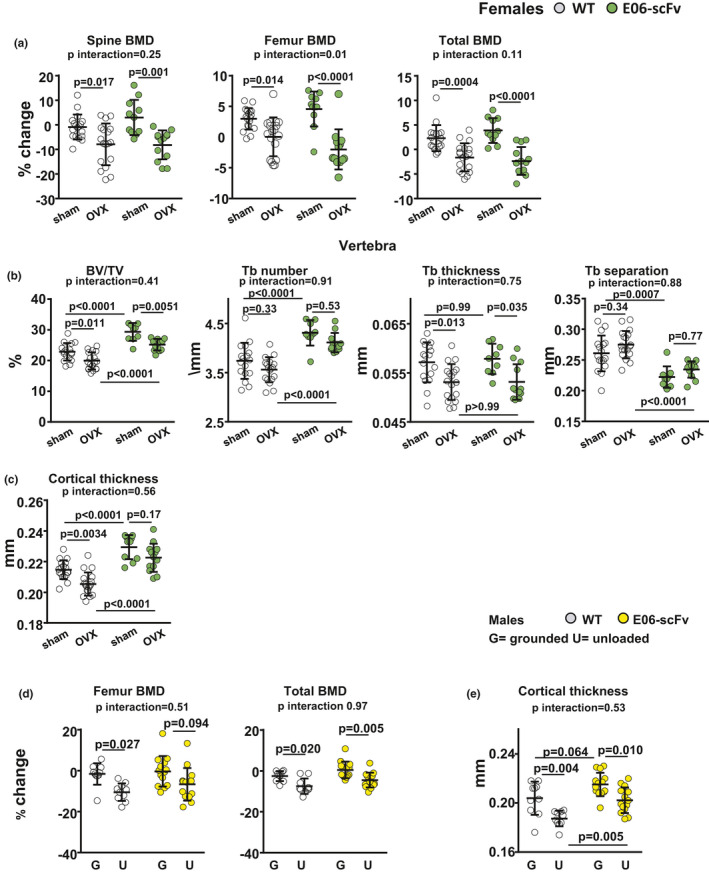
E06‐scFv does not attenuate OVX‐ or unloading‐induced bone loss. a‐c, WT and E06‐scFv transgenic mice (4.5 months old) were ovariectomized or sham‐operated and euthanized 6 weeks later. a, Femoral, spinal, and total BMD by DXA were performed five days before surgery and three days before euthanasia; results are expressed as % change from baseline BMD. WT *n* = 18; E06‐scFv *n* = 11–13. Adjusted *p*‐values were calculated by repeated measures using two‐way ANOVA. b, Quantification of bone architecture by micro‐CT in vertebrae (trabecular bone) [WT *n* = 17–18; E06‐scFv *n* = 9–12]. c, Cortical thickness at the femoral diaphysis (cortical bone) [WT *n* = 18; E06‐scFv *n* = 11–13]. Data are analyzed by two‐way ANOVA, the shown *p*‐values are adjusted using the Sidak's multiple comparison procedure. BV/TV = bone volume/total volume; Tb = trabecular. d‐e, The posterior limbs of 5‐month‐old WT and E06‐scFv transgenic male mice were unloaded for 3 weeks; control group mice were left grounded. d, Femoral and total BMD by DXA were performed three days before unloading and on the day of euthanasia; results are expressed as % change from baseline BMD [WT *n* = 10; E06‐scFv *n* = 14]. Adjusted *p*‐values were calculated by repeated measures using two‐way ANOVA. e),Cortical thickness by micro‐CT in femoral diaphysis of the same mice as in a). Data are shown as individual values including mean and standard deviation. Data were analyzed by two‐way ANOVA, the shown *p*‐values were adjusted using Sidak's multiple comparison procedure

Four‐and‐a‐half‐month‐old WT and E06‐scFv transgenic mice were ovariectomized or sham‐operated and euthanized 6 weeks later. After OVX, both WT and E06‐scFv transgenic mice exhibited the expected increase in body weight (Supplementary Figure 2A) and loss of uterine weight (Supplementary Figure 2B). OVX caused a similar decrease in spinal, femoral, and total BMD in both WT and E06‐scFv transgenic mice (Figure 5A). After OVX, vertebral trabecular bone decreased by 12.8% in WT and 14.1% in E06‐scFv transgenic mice (*p* interaction 0.41) (Figure 5B). The loss of bone was secondary to loss of trabecular thickness, as trabecular number and separation were similar between sham and OVX mice in both genotypes. After OVX, we did not observe a decrease in the trabecular bone of the femur in WT mice (Supplementary Figure 2C). The transgenic mice, however, lost bone at this site, likely because of their higher starting bone mass, which enabled the detection of a decrease in bone mass. At the femoral diaphysis, WT mice exhibited a 4.3% decrease in cortical thickness after OVX, while E06‐scFv transgenic mice lost 3.0% of bone at the same site (*p* interaction 0.56) (Figure 5C).

To investigate whether E06‐scFv protects against unloading‐induced bone loss, the posterior limbs of 5‐month‐old male WT and E06‐scFv transgenic mice were unloaded for 3 weeks. WT and transgenic mice lost a comparable amount of weight during the experiment (8% and 10.9%, respectively) (Supplementary Figure 3A). Unloading caused a similar decrease in femoral and total BMD in both WT and E06‐scFV transgenic mice (Figure 5D). Similarly, WT and E06‐scFv transgenic mice exhibited a comparable loss of femoral cortical thickness of 8.1% and 6.0%, respectively (Figure 5E). There were no changes in trabecular bone volume or microarchitecture at the femoral metaphysis in either WT or transgenic mice (Supplementary Figure 3B). Although there was a decrease in trabecular thickness in WT mice, the significance of this observation is unclear because there was no change in bone volume.

### 2.6 E06‐scFv increases Wnt signaling in young and old mice

We began to search for the molecular mechanisms by which neutralization of OxPLs attenuates age‐associated trabecular bone loss. Notably, cellular senescence has been implicated in skeletal aging (Khosla et al., 2020). To identify whether E06‐scFv expression affected senescence, we measured the RNA expression of three markers of senescence in vertebral bone: cells cycle inhibitors p21 and p16 (Childs et al., 2015; Munoz‐Espin & Serrano, 2014) and NADase CD38 (Chini et al., 2020; Covarrubias et al., 2020). We found that only p16 expression increased in aged mice; nevertheless, E06‐scFv did not alter its expression (Supplementary Figure 4A).

We also examined the mRNA levels of the two genes critical for osteoclastogenesis: *RANKL* and *OPG*. In vertebral bone, E06‐scFv increased the expression of RANKL in both young and old mice, a change that did not explain the decrease in osteoclast number in these animals (Supplementary Figure 4B). We could not detect a change in OPG expression between the two genotypes (Supplementary Figure 4C).

**FIGURE 6:**
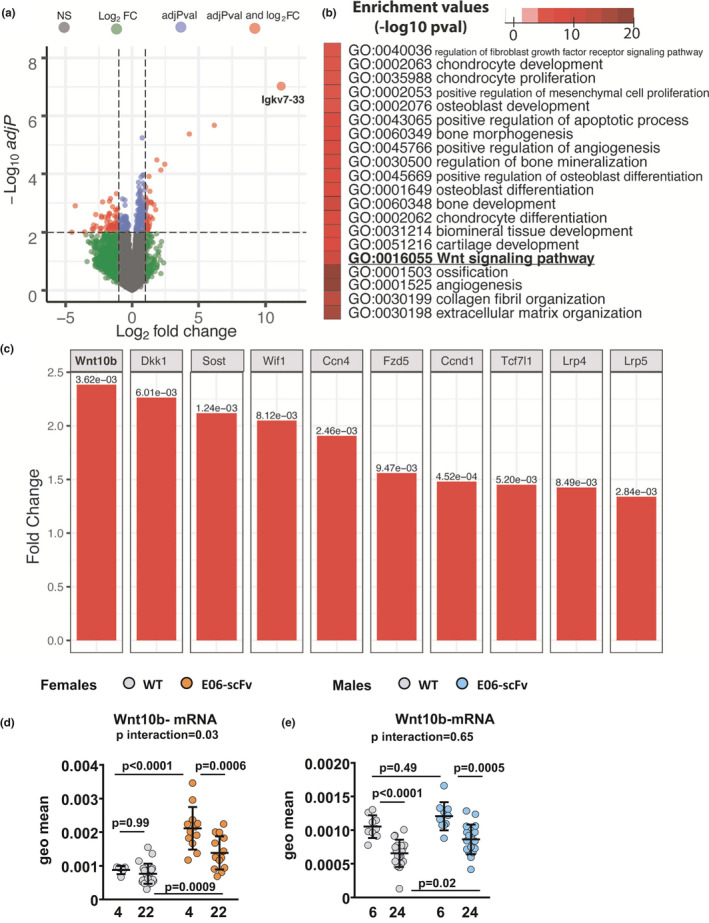
E06‐scFv increases Wnt signaling in the vertebrae. Transcriptome analysis of vertebrae comparing 22‐month‐old E06‐scFv transgenic mice and WT controls. a, Volcano plot of differential gene expression analysis. Solid circles represent the log2fold changes and statistical adjusted *p* values of individual genes. The vertical dashed lines represent the log2fold changes of 1, and the horizontal line represents an adjusted *p* values cutoff of 0.01. Gray, green, blue, and red circles represent genes that did not pass the cutoff, only passed the log2fold change cutoff, only passed the adjusted *p*‐values cutoff, or passed both cut‐offs, respectively. The most upregulated gene Igkv7‐33 is illustrated as the largest orange circle. b, Heat map of selected upregulated functional enrichment analysis of GO terms related to bone‐related processes, including Wnt signaling pathway. Complete results of the functional enrichment analysis are provided in Supplementary Figure 5. c, Bar plots of fold changes and adjusted *p*‐values of differentially expressed genes (fold change >1.25 and adjusted *p*‐value <0.01) in Wnt signaling pathway [WT *n* = 5; E06‐scFv *n* = 5]. d, Gene expression of Wnt10b in vertebral bone of 4‐month‐old and 22‐month‐old WT and E06‐scFv transgenic female mice [WT *n* = 5–22; E06‐scFv *n* = 12–14]. e) Gene expression of Wnt10b in vertebral bone of 6‐month‐old and 24‐month‐old WT and E06‐scFv transgenic male mice [WT *n* = 9–19; E06‐scFv *n* = 10–18]. Transcripts were normalized to each of 5 housekeeping genes (ChoB, ActB, B2 M, 18S, and Hrpt1), and the transcript levels were calculated as the geometric mean of the 5 normalized values

We then used an unbiased approach and performed RNA‐seq analysis to compare the transcriptome of vertebrae between aged WT controls and E06‐scFv transgenic female mice. With an adjusted p‐value cutoff of 0.05, we identified 1179 differentially expressed genes (506 upregulated and 670 downregulated). As shown in the volcano plot (Figure 6A), the most significantly upregulated gene was immunoglobulin kappa chain variable 7–33 (Igkv7‐33), which is 98% identical to the E06‐scFv transgene sequence. This confirmed the high expression of the transgene in E06‐scFv mice. We further investigated the impact of the transgene on cellular processes based on gene ontology annotation using gene set enrichment analysis (Väremo et al., 2013). In line with the evidence that the transgene increases the osteoblast number in bone, E06‐scFv upregulated many osteoblast‐related processes, including osteoblast differentiation, mesenchymal cell proliferation, bone mineralization, and extracellular matrix organization. The transgene also upregulated the Wnt signaling pathway (Figure 6B), which is known to be a key factor for the differentiation and survival of osteoblasts (Baron & Kneissel, 2013). Specifically, the RNA‐seq analysis revealed that E06‐scFv upregulated Wnt inhibitors (Dkk1, Sost, and Wif1), Wnt target genes [Ccnd4 (Wisp1) and Ccnd1 (CyclinD1)], Wnt receptors (Fzd5, Lrp4 and Lrp5), and the transcription factor Tcf7l1, which is activated by beta‐catenin and mediates the Wnt signaling pathway. Interestingly, the most upregulated gene in the Wnt signaling pathway was wingless‐type MMTV integration site family member 10B (Wnt10b) (Figure 6C).

Gene expression analysis by qPCR in the vertebrae of female mice confirmed the increase in Wnt10b in 22‐month‐old transgenic females compared with WT mice of the same age (Figure 6D). The increase in Wnt10b expression in vertebral bone was also seen in 4‐month‐old E06‐scFv homozygous female mice (Figure 6D) and in a separate group of 6‐month‐old hemizygous and homozygous E06‐scFv transgenic females, in which we previously found increased bone mass (Palmieri et al., 2021) (Supplementary Figure 6). We could not detect an increase in Wnt10b between WT and E06‐scFv young male mice (Figure 6E). Nevertheless, Wnt10b remained elevated in aged E06‐scFv male mice compared with WT littermates despite the decline with age in both genotypes (Figure 6D). The increase in Wnt10b and the activation of canonical Wnt signaling could contribute to increased osteoblasts and increased bone formation in E06‐scFv transgenic mice (Bennett et al., 2005, 2007).

## 3 DISCUSSION

The results presented in this report indicate that targeting oxidized phospholipids, by overexpressing the E06‐svFv transgene, attenuates the age‐associated trabecular bone loss in both female and male mice. Therefore, OxPLs not only have an adverse effect on bone mass during the first 6 months of life in mice, but contribute to age‐related bone loss, presumably due to both a continued increase in oxidative stress and generation of OxPLs, together with a decrease in natural anti‐PC antibodies (Almeida et al., 2009; Ambrogini et al., 2018; Barrera et al., 2018; Manolagas, 2010). Importantly, similar pathogenic mechanisms may contribute to other age‐associated diseases such as atherosclerosis (Binder et al., 2016; Que et al., 2018), cardiovascular disease (Byun et al., 2017; Capoulade et al., 2018; Tsimikas et al., 2012), steatohepatitis (Sun et al., 2019), macular degeneration (Handa et al., 2015; Suzuki et al., 2012), and Alzheimer's dementia (Ademowo et al., 2017; Binder et al., 2016).

The results of the present work also indicate that aged E06‐scFv mice maintain higher trabecular bone mass than WT littermates because of increased osteoblasts and decreased osteoclasts throughout adult life.

In mice, the age‐associated loss of bone in the trabecular compartment is characterized by low remodeling and a decrease in osteoblast number and bone formation rate, as well as a decrease in osteoclast number (Almeida et al., 2007). Importantly, one of the histological hallmarks of aged bone in humans is decreased wall width, a reliable measure of the amount of bone made by a team of osteoblasts (Lips et al., 1978; Manolagas, 2010). The mechanism responsible for the decline of osteoblast number in aging bone is largely unknown. Aging decreases the number of mesenchymal stem/progenitors cells, and this process in associated with increased markers of senescence (Kim et al., 2017). In our studies, aging decreased the osteoblast number in both genotypes, indicating that mechanisms other than OxPLs are responsible for this decline. Indeed, we found that p16, a marker of cell senescence, increased at a similar rate in both genotypes. Nevertheless, E06‐scFv maintained a higher number of osteoblasts in aged mice.

The mechanisms by which E06‐scFv increases osteoblast number remain unclear. Products of lipid peroxidation, such as 4‐Hydroxynonenal (4‐HNE) and PC‐OxPL, decrease proliferation, inhibit differentiation, and stimulate apoptosis of osteoblasts *in vivo* and *in vitro* (Almeida et al., 2009; Ambrogini et al., 2018; Palmieri et al., 2021). The results of the RNA‐seq analysis in aged E06‐scFv transgenic mice suggest that E06‐scFv expression upregulates Wnt10b and Wnt target genes. These findings indicate that neutralizing OxPLs increases Wnt signaling *in vivo* and are consistent with several lines of earlier evidence linking the adverse effects of OxPLs with inhibition of Wnt signaling (Almeida et al., 2009; Liu et al., 2016; Wang et al., 2018; Ye et al., 2016). Specifically, increased lipoxygenase expression, which increases lipid oxidation, reduces canonical Wnt signaling in calvaria and vertebrae of 4‐month‐old mice (Almeida et al., 2009), and oxidized polyunsaturated fatty acids attenuate the proliferation of osteoblastic cells and the differentiation of bone‐marrow‐derived osteoblast progenitors induced by Wnt3a (Almeida et al., 2009). In addition, the increase in OxPLs with a high‐fat diet reduces the expression of several of the same genes that are upregulated by E06‐scFv in the vertebrae, such as the Wnt ligand Wnt10b, the Wnt antagonists Dkk1 and Sost, and the Wnt target Wisp1 (Ccn4) (Liu et al., 2016).

Wnt10b is one of the 19 known Wnt ligands and stimulates osteoblastogenesis through activation of the canonical Wnt signaling pathway (Bennett et al., 2005, 2007). Wnt10b decreases in the femoral cortical bone of female mice between 7 and 21 months of age (Piemontese et al., 2017). Transgenic mice overexpressing Wnt10b in the bone marrow and adipose tissue have increased trabecular bone and are protected against age‐associated bone loss. In addition, transgenic mice overexpressing Wnt10b under the control of the osteocalcin promoter have increased trabecular bone in the femur due to an increase in mineral apposition rate, mineralizing surface, and bone formation rate, without changes in osteoclasts (Bennett et al., 2007). Conversely, global deletion of Wnt10b decreases trabecular bone (Bennett et al., 2005) and bone formation rate (Bennett et al., 2007). Wnt10b affects osteoblast differentiation by inducing Runx2, Dlx5, and Osterix and maintaining mesenchymal progenitor activity (Stevens et al., 2010), but not proliferation (Bennett et al., 2007). The similarities between the bone phenotype in the E06‐scFv transgenic mice and the Wnt10b overexpression model support the notion that Wnt10b is a mediator of the increased osteoblastogenesis seen in our model. The cellular source of Wnt10b remains unknown, but Wnt10b is expressed in pre‐adipocytes, stromal vascular cells (Bennett et al., 2002), bone marrow, the postnatal growth plate (Andrade et al., 2007), and osteoblastic precursors (Zhou et al., 2008). Genetic manipulation of Wnt10b in our mouse model will likely further clarify this mechanism.

Activation of canonical Wnt signaling in osteoclast precursors suppresses osteoclast differentiation and increases osteoclast apoptosis (Albers et al., 2013; Otero et al., 2012; Ruiz et al., 2016). However, Wnt10b does not affect osteoclastogenesis. We could not detect changes in Wnt ligands that alter osteoclastogenesis, such as Wnt16, Wnt4, and Wnt5a (Maeda et al., 2019). In addition, we did not detect changes in the expression of RANKL or OPG that could explain the anti‐osteoclastogenic effect of the transgene. Therefore, the cause of the decrease in osteoclasts in the vertebral bone of E06‐scFv transgenic mice remains to be determined. The current knowledge on the role of OxPLs in osteoclastogenesis is somewhat controversial and often incongruent. OxPLs stimulate the production of osteoclastogenic cytokines and osteoclastogenesis *in vitro* (Tintut et al., 2002; Tseng et al., 2010). OxLDL has been shown both to promote (Graham et al., 2009; Maziere et al., 2013) and decrease osteoclastogenesis *in vitro* (Maziere et al., 2009), and high‐fat diet decreases osteoclasts *in vivo* (Y. Liu et al., 2016). Therefore, future work is needed to elucidate this mechanism.

Unlike the effects on trabecular bone loss, E06‐scFv did not attenuate the age‐associated loss of cortical bone or the increase in cortical porosity. An increase in osteoclast number and bone resorption are critical components of age‐related cortical bone loss and porosity (Li et al., 2015; Piemontese et al., 2017; Ucer et al., 2017). The increase in osteoclasts is associated with increased expression of RANKL by senescent osteocytes (Kim et al., 2020), and blocking RANKL completely prevents cortical bone loss in aged mice (Kim et al., 2020). Our previous work showed that E06‐scFv does not alter the osteoclast number at the endocortical surface (Ambrogini et al., 2018; Palmieri et al., 2021). Therefore, the lack of protection against the age‐related cortical bone loss with the E06‐scFv transgene could be explained by the fact that E06‐scFv does not prevent the age‐related increase of osteoclasts at endosteal and intracortical sites.

Expression of the E06‐scFv did not attenuate the bone loss induced by OVX or unloading. An increase in osteoclast number and bone resorption are critical components of the loss of bone in these two models (Almeida et al., 2017; Xiong et al., 2011). Our findings suggest that OxPLs play no role in the pathogenesis of these two conditions and that the increased bone formation due to E06‐scFv is not sufficient to counteract the increase in osteoclast number. Despite the fact that E06‐scFv did not attenuate the loss of cortical bone with aging, OVX, and unloading, the E06‐scFv transgenic mice had increased bone mass compared with aged or similarly treated WT mice, suggesting that E06‐scFv may still exert its anabolic activity in these conditions. Future studies with E06‐scFv administration should elucidate this possibility.

In closing, the results reported herein are consistent with the hypothesis that with aging there is increased lipid peroxidation and the generation of oxidized phospholipids, which contribute to the age‐associated trabecular, but not cortical, bone loss in mice. The age‐related decline in innate antibodies targeting OxPLs likely contributes as well. Because OxPLs are in large part products of non‐enzymatic lipid peroxidation and constitute a wide variety of OxPL moieties, it is difficult to imagine generalized strategies to neutralize their adverse effects. However, the discovery that innate natural antibodies targeting the PC head group of OxPLs, such as E06, neutralize their adverse effects (Ambrogini et al., 2018; Binder et al., 2016; Palmieri et al., 2021; Que et al., 2018) suggests a way to target OxPLs *in vivo*. The development of natural anti‐PC antibodies that mimic the properties of E06, therefore, represents a novel therapeutic approach to countering the accumulation of PC‐OxPLs with age and their adverse effects in osteoporosis, as well as other age‐associated diseases such as atherosclerosis and non‐alcoholic steatohepatitis. Notably, blocking OxPL increases Wnt10b and canonical Wnt signaling in bone, providing a novel mechanistic explanation of the bone‐anabolic efficacy of the E06‐scFv transgene.

## 4 MATERIALS AND METHODS

### 4.1 Animals

E06‐scFv transgenic mice have been described previously (Ambrogini et al., 2018; Palmieri et al., 2021; Que et al., 2018; Sun et al., 2019). The E06‐scFv transgene produces a fusion protein of the heavy and light chain of the variable region domains of the E06 IgM antibody, joined by a flexible peptide linker; it is under the control of the ApoE promoter (Que et al., 2018). For the experiments described in this paper, hemizygous E06‐scFv transgenic mice in the C57BL/6 background were bred to obtain wild‐type (WT) and homozygous littermates. As previously described (Ambrogini et al., 2018), E06‐scFv copy number was measured in tail extracts using qPCR of genomic DNA and used to genotype the mice. Mice were group‐housed in our vivarium at a constant temperature of 23℃, with a 12:12‐hour light‐dark cycle under specific pathogen‐free conditions. Mice had ad libitum access to water and diet. Breeding and experimental mice were fed with chow rodent diet [Teklad 22/5 (8640), Envigo].

### 4.2 Aging experiment

At 6 months of age, mice were switched to Teklad Global 14% Rodent Maintenance Diet (2014), Envigo. Female and male mice were euthanized at 22 and 24 months of age, respectively. Separate cohorts of 4‐month‐old female and 6‐month‐old male mice were euthanized at the same time as the aged mice as young controls. For dynamic histomorphometric analysis, the mice were injected intraperitoneally with calcein (20 mg/kg body weight; Sigma‐Aldrich), 8 and 2 days before the harvest. During the course of the experiment, in the female WT group, 2 mice were euthanized for severe dermatitis at 12 and 19 months, and 1 mouse was found unresponsive with severe dermatitis and was euthanized at 20 months. In the E06‐svFv female group, 3 mice were found dead with no obvious cause at the age of 17, 19, and 21 months; 2 mice were euthanized for extensive corneal ulcerations at 18 and 20 months; 4 mice were euthanized for severe dermatitis at 11, 17 (2 mice), and 20 months; 1 mouse was euthanized for malocclusion at 13 months; and 2 mice were euthanized for severe exophthalmos at 16 and 18 months. Four mice died during technical procedures at the end of the experiment. In the male WT group, 6 mice were found dead without obvious cause (1 at 15 months, 1 at 21 months, 1 at 23 months, and 3 at 24 months). Four mice were euthanized following the detection of tumors (abdominal masses at the age of 22 months [2 mice] and 24 months [1 mouse] and a neck mass [1 mouse] at 18 months); 1 mouse was euthanized for severe dermatitis at 23 months. Three mice died at 24 months after technical procedures. In the male E06‐scFv group, 1 mouse was euthanized at 18 months for bilateral adrenal tumors, 1 mouse was euthanized at 17 months for severe injury to the prepuce, and 1 mouse was euthanized at 24 months for dermatitis. Five mice died of no apparent cause, 1 at 23 months and 4 at 24 months.

### 4.3 Ovariectomy

Eighteen‐week‐old WT and E06‐scFv homozygous transgenic littermates were randomized into sham operation or OVX groups based on their femoral BMD measured by DXA. BMD and weight measurements were obtained 5 days before surgery and 3 days before the harvest. Mice were euthanized 6 weeks postsurgery, and tissues were collected for analysis. Uterine weight was measured soon after dissection.

### 4.4 Unloading

Mechanical unloading of hindlimbs was performed by tail suspension of 5‐month‐old WT and E06‐scFv homozygous transgenic male littermates for 21 days. The mice were randomized to the unloaded or grounded (control) group according to their femoral BMD. BMD and body weight were obtained 3 days before tail suspension and on the day of euthanasia. Hindlimb suspension was performed as previously described (Xiong et al., 2011). Briefly, the mouse tail was wrapped with medical tape (leaving the tip of the tail exposed to check for discoloration and necrosis) and attached to a straightened paper clip connected to a fishing swivel by a 2.5‐cm‐long hobby chain. The hindlimbs were suspended by connecting the fishing swivel to a key ring inserted in a rod placed on the top of a mouse cage. The mouse was maintained with a ~30° head‐down tilt. One tail‐suspended and one grounded animal were housed per cage (separated by a plastic divider). The animals were euthanized after 3 weeks, and the tissues were collected for analysis.

In both the OVX and unloading experiments, the mice were injected intraperitoneally with calcein (20 mg/kg body weight; Sigma‐Aldrich) 7 and 3 days before harvest.

In all the experiments, the animals were euthanized by CO_2_ inhalation.

### 4.5 Bone imaging

Skeletal architecture was assessed with dual‐energy X‐ray absorptiometry (DXA) and micro‐CT. BMD measurements by DXA were performed using a PIXIimus densitometer (GE Lunar) in mice sedated with 2% isoflurane, as previously described (O'Brien et al., 2005). The mean coefficient of BMD variation, calculated during the conduct of these studies prior of each use using a proprietary phantom, was 0.43%. Micro‐CT measurements were obtained using a micro‐CT40 scanner (Scanco Medical AG) as previously described (Palmieri et al., 2021). Briefly, the fifth lumbar vertebra (L5) and left femur were dissected and cleaned of soft tissues. The left femur was placed in 10% Millonig's Neutral Buffered Formalin with 5% sucrose fixative (Leica Biosystems Inc.), and L5 was placed in B‐plus fixative (BBC Biomedical). After fixation, bones were dehydrated with repeated passages in progressively increasing concentrations of ethanol and kept in 100% ethanol until analysis. The micro‐CT scanning and analysis for trabecular bone at the vertebra and femur and the cortical bone at the femoral diaphysis (midpoint of the bone length as determined at scout view) were performed as previously described (Ambrogini et al., 2018; Palmieri et al., 2021). Cortical porosity at the femur was measured from the diaphysis to an area immediately adjacent to the primary spongiosa, as previously described (Piemontese et al., 2017). While performing these studies, the mean coefficient of variation of the micro‐CT phantom was monitored weekly and was 0.0054%.

### 4.6 Histomorphometry

After dissection, L1‐L3 vertebrae were placed in 10% Millonig's Neutral Buffered Formalin with 5% sucrose fixative, dehydrated with ethanol, kept in ethanol 100% until analysis, and embedded in methyl methacrylate (Sigma‐Aldrich). Static and dynamic histomorphometric determinations were made as previously described (Ambrogini et al., 2018; Palmieri et al., 2021). Briefly, for osteoblast and osteoclast number and surface, the sections were stained with toluidine blue and TRAP as previously described (Ambrogini et al., 2018; Palmieri et al., 2021). Osteoblast and osteoclast numbers were reported over the bone perimeter. Unstained sections were used for the determination of fluorescent calcein labeling using fluorescence microscopy. The measurements were performed by an operator blinded to the identity of the samples using Osteomeasure version 7 V4.3.0.0 (OsteoMetrics Inc.). Histomorphometric data are reported using the nomenclature recommended by the American Society for Bone and Mineral Research (Dempster et al., 2013).

### 4.7 Quantitative PCR (qPCR)

Total RNA was extracted from vertebral bone with TRIzol (Thermo Fisher Scientific) and purified with Direct‐zol RNA Miniprep (Zymo Research, cat no. R2050) or a Qiagen RNeasy Plus Mini Kit (Qiagen) according to the manufacturer's instructions. Complementary DNA (cDNA) was reverse‐transcribed from 0.5 µg of total RNA extract using the High‐Capacity cDNA Reverse Transcription Kit (Applied Biosystems) according to the manufacturer's instructions. TaqMan real‐time PCR was performed using TaqMan Gene Expression Assays manufactured by Applied Biosystems, as listed in Supplementary Table 1. Transcripts were normalized to each of 5 housekeeping genes (*ChoB*, *ActB*, *B2 M*, *18S*, *and Hrpt1*), and the transcript levels were calculated as the geometric mean of the 5 normalized values.

### 4.8 RNA‐seq library preparation

Total RNA was isolated from mouse vertebrae with the Qiagen RNeasy Plus Mini Kit (Qiagen) according to the manufacturer's instructions. RNA‐seq library preparation was performed by Novogene (Novogene Corporation Inc.).

### 4.9 RNA‐seq analysis

The raw FASTQ files were preprocessed and analyzed as previously described (Jenjaroenpun et al., 2018). In brief, the raw FASTQ files were quality trimmed to obtain the high‐quality reads using SolexaQA++ software (Cox et al., 2010). The high‐quality reads were mapped to the reference mouse genome mm10 using BWA software (Li & Durbin, 2009), and then, SAMtools (Li et al., 2009) was used to generate BAM files. The BAM files were used to calculate gene expression count for individual samples using bedtools2 (Quinlan & Hall, 2010). The count table was used for differential gene expression analysis in transgenic versus control mice with the voom method (Law et al., 2014). The raw FASTQ files were deposited to the Sequence Read Archive at the National Center for Biotechnology Information under BioProject accession number PRJNA704398.

### 4.10 Statistics

No experimentally derived data were excluded. Poor section quality precluded the histological analysis of the bone formation rate for some samples (Figure 4). Each figure legend includes the number of mice analyzed in the experiments. All data were collected and analyzed by personnel blinded to the identity of the samples. Single data points including mean ±standard deviation are shown in every figure except Figure 1 where data are shown as mean ±standard deviation only.

Statistical analysis for the data shown in Figure 1 was performed using R (version 3.5). Statistical analyses of the data shown in the other figures were performed using GraphPad Prism (versions 7.0.4 and 8.0.1). Group mean values were compared by one‐way or two‐way ANOVA or repeated measures ANOVA, as appropriate. Pairwise multiple comparisons were performed and the p‐values adjusted using the Tukey's pairwise comparison procedure or the Holm–Sidak method.

For RNA‐seq analysis in Figure 6, the p‐value of each individual gene was corrected for multiple testing using the Benjamini–Hochberg method to generate adjusted p‐values. PIANO package (Väremo et al., 2013) was used to perform the gene set analysis of Gene Ontology (GO). We selected the GO terms that had adjusted enrichment *p*‐value less than 10e‐5 and plotted a heatmap that represents the results of functional analysis.

For *in vivo* studies, the sample sizes used were adequate to detect a difference of 1.2 standard deviations at a power of 0.8 and *p* < 0.05 (Liu et al., 2016).

### 4.11 Study approval

All animal procedures were approved by the Institutional Animal Care and Use Committees of the University of Arkansas for Medical Sciences and the Central Arkansas Veterans Healthcare System.

## CONFLICT OF INTEREST

ST, XQ, and JLW are co‐inventors and receive royalties from patents owned by the University of California, San Diego (UCSD) on oxidation‐specific antibodies and of biomarkers related to oxidized lipoproteins and are co‐founders and have an equity interest in Oxitope, Inc. and its affiliates (“Oxitope”). ST and JLW are co‐founders of Kleanthi Diagnostics, LLC. Although these relationships have been identified for conflict of interest management based on the overall scope of the project and its potential benefit to Oxitope and Kleanthi, the research findings included in this particular publication may not necessarily relate to the interests of Oxitope and Kleanthi. The terms of this arrangement have been reviewed and approved by UCSD in accordance with its conflict of interest policies. ST has a dual appointment at UCSD and Ionis Pharmaceuticals and is a consultant to Boston Heart Diagnostic. JLW is a consultant to Ionis Pharmaceuticals. All other authors declare no competing financial interests.

## AUTHOR CONTRIBUTIONS

EA, SCM, JLW, and MA developed the concept, designed the experiments, and analyzed the data. MP, TJ, and XS performed the experiments. IN processed and analyzed the RNA‐seq data. HGA performed part of the statistical analysis. XQ generated the E06‐scFv transgenic mice, and ST provided important critiques. EA performed the analyses, created the figures, and wrote the first draft of the manuscript with subsequent contributions from all authors, who commented on it at all stages.

## Supporting information

Fig S1

Fig S2

Fig S3

Fig S4

Fig S5

Fig S6

Table S1

## Data Availability

The datasets generated and/or analyzed during the current study are available from the corresponding author upon reasonable request.
