# Supplementary figures and images for: Neutralization of oxidized phospholipids attenuates age‐associated bone loss in mice
Source: Aging Cell. 2021 Jul 19;20(8):e13442. doi: 10.1111/acel.13442 (PMC8373359; doi:10.1111/acel.13442)

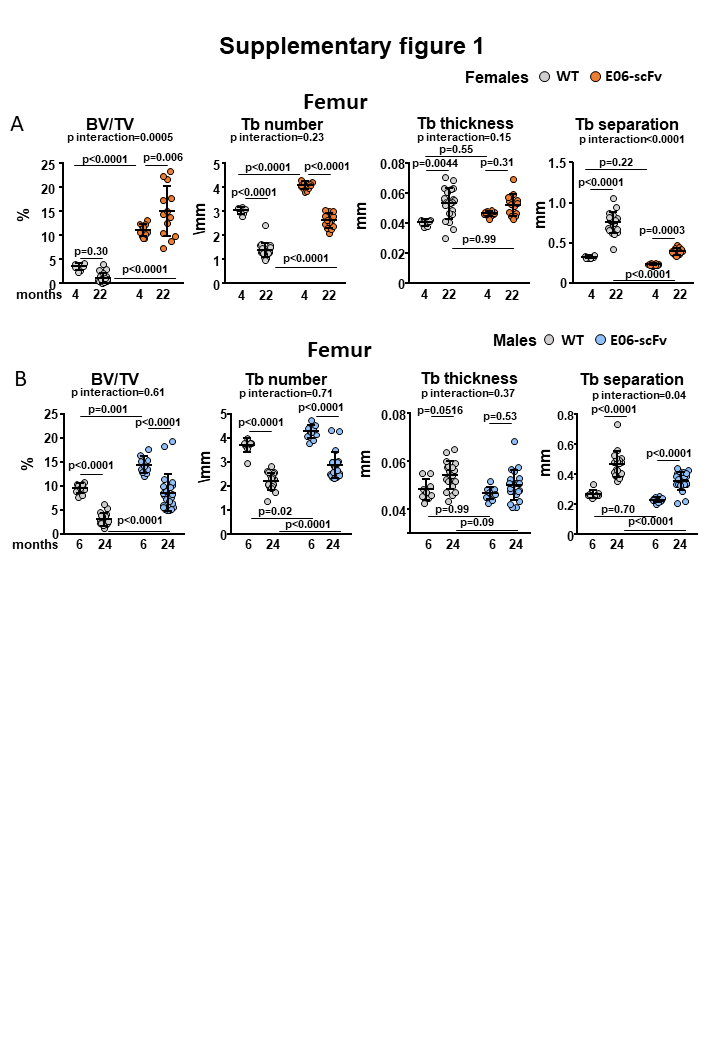

Supplement: Supplementary file 1 — Fig S1 [file ACEL-20-e13442-s001.tif]

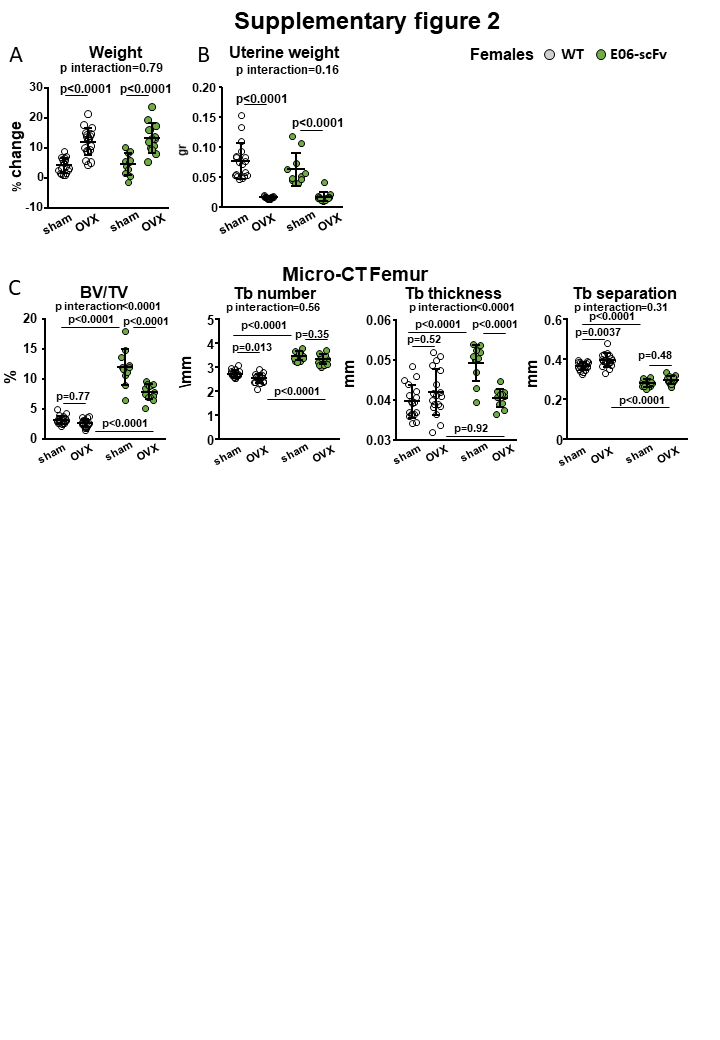

Supplement: Supplementary file 2 — Fig S2 [file ACEL-20-e13442-s002.tif]

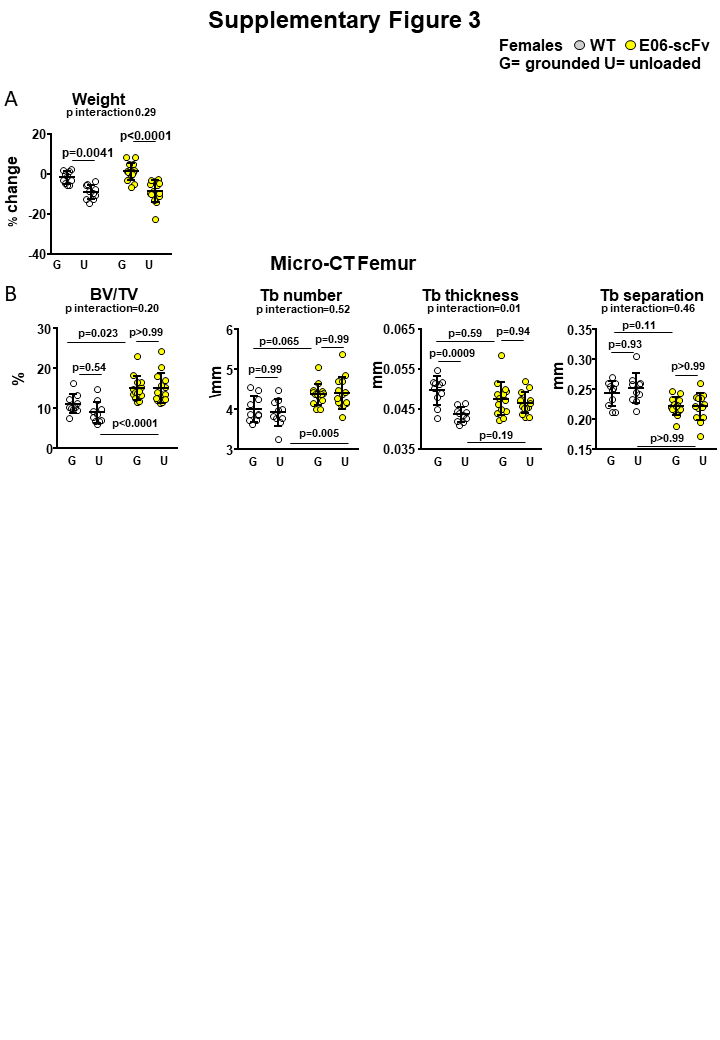

Supplement: Supplementary file 3 — Fig S3 [file ACEL-20-e13442-s007.tif]

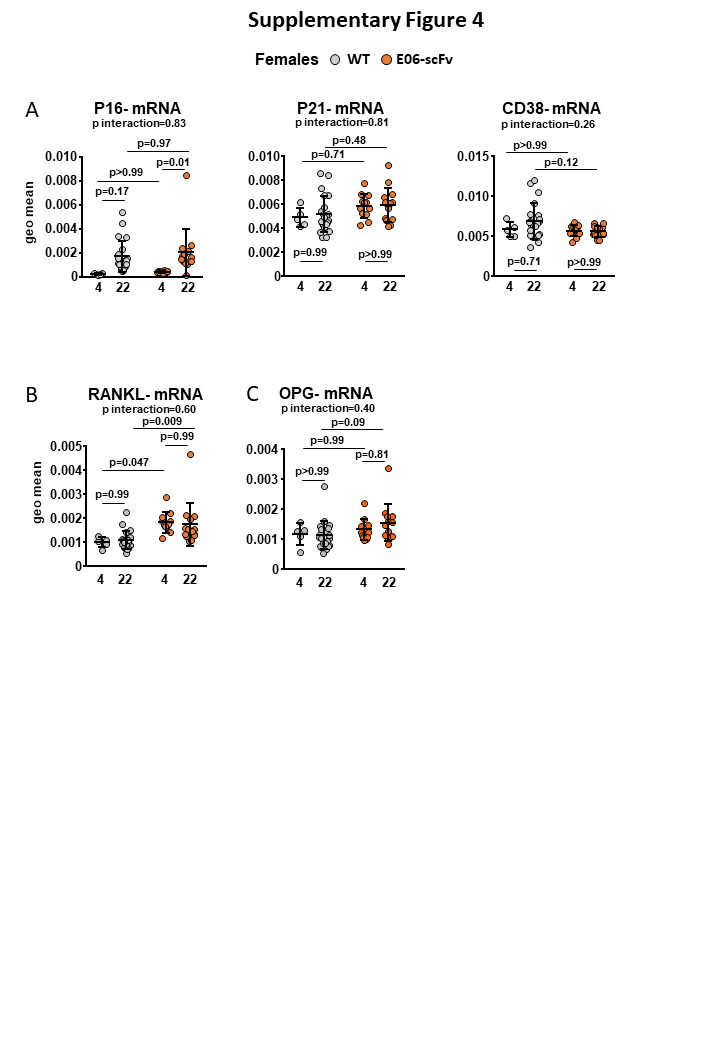

Supplement: Supplementary file 4 — Fig S4 [file ACEL-20-e13442-s003.tif]

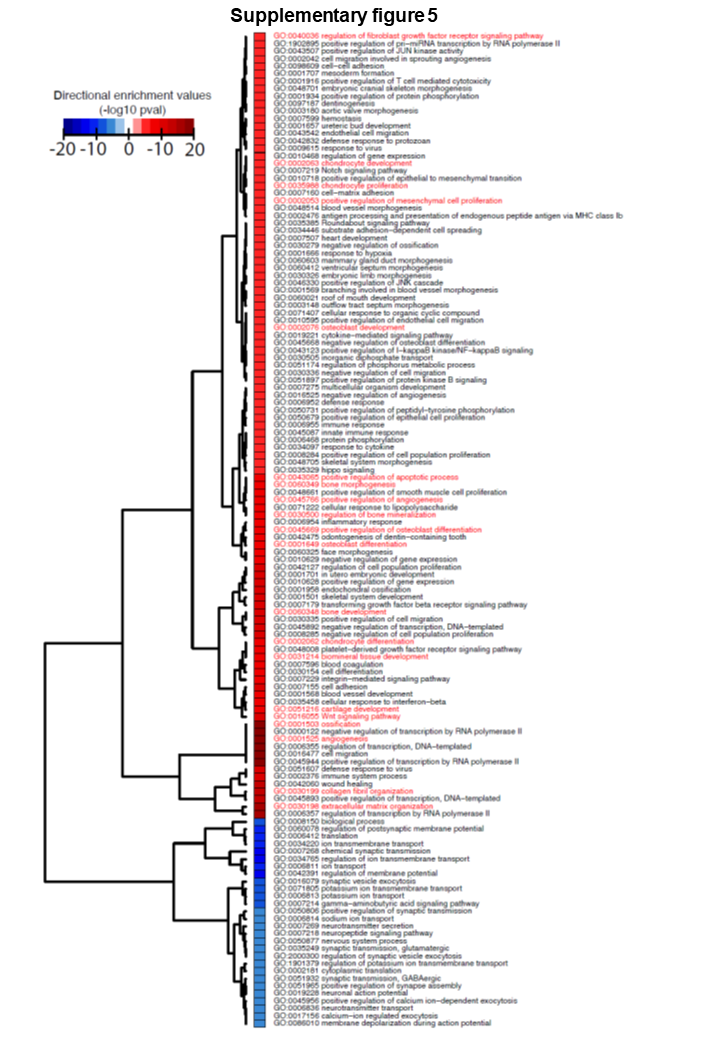

Supplement: Supplementary file 5 — Fig S5 [file ACEL-20-e13442-s006.tif]

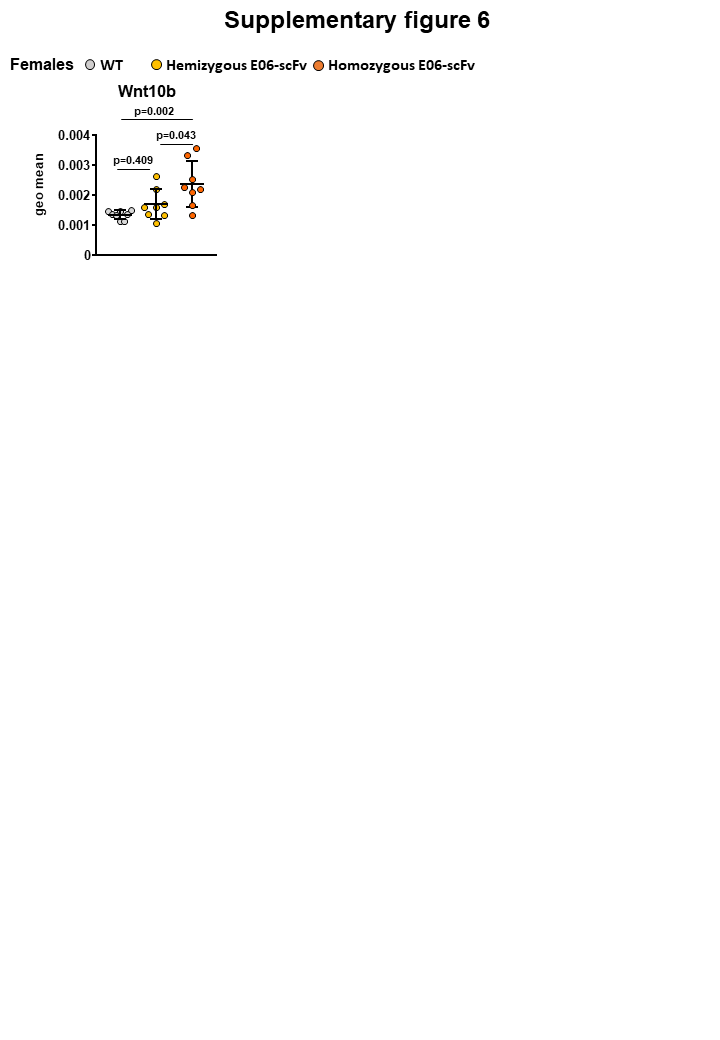

Supplement: Supplementary file 6 — Fig S6 [file ACEL-20-e13442-s004.tif]

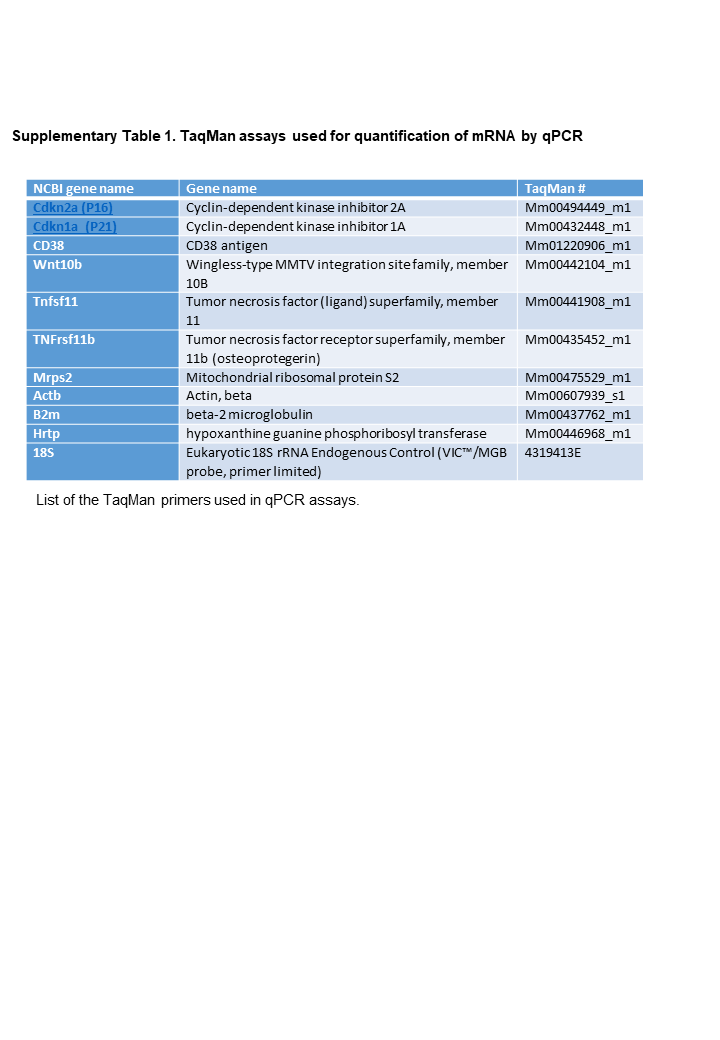

Supplement: Supplementary file 7 — Table S1 [file ACEL-20-e13442-s005.tif]
